# Mechanical Regulation of Bone Regeneration: Theories, Models, and Experiments

**DOI:** 10.3389/fendo.2014.00211

**Published:** 2014-12-10

**Authors:** Duncan Colin Betts, Ralph Müller

**Affiliations:** ^1^Institute for Biomechanics, ETH Zürich, Zürich, Switzerland

**Keywords:** bone regeneration, fracture healing, mechanobiology, simulation

## Abstract

How mechanical forces influence the regeneration of bone remains an open question. Their effect has been demonstrated experimentally, which has allowed mathematical theories of mechanically driven tissue differentiation to be developed. Many simulations driven by these theories have been presented, however, validation of these models has remained difficult due to the number of independent parameters considered. An overview of these theories and models is presented along with a review of experimental studies and the factors they consider. Finally limitations of current experimental data and how this influences modeling are discussed and potential solutions are proposed.

## Introduction

Bone’s capability of “perfect” regeneration is unique, unlike other tissues it is capable of recovering its form without permanent scars. However not all fractures heal spontaneously, it has been found that 20 per 100,000 people per year will have delayed healing or a non-union, where the fractured bone fails to fuse (1). It is known that mechanical forces can influence the pathways through which healing occurs; several studies have shown that changes in the mechanical environment can modulate the time taken to heal, change the proportions of different tissue type as well as gene expression patterns of cells in the healing bone (2, 3). The exact mechanism through which mechanical stimuli are sensed and incorporated in the healing process is not fully understood and still remains an open question. Answering this question will lead to improved treatment methods for bone fracture repair, reducing the amount of time patients are hospitalized. To this end, we have compiled a summary of literature on the topic examining the experimental studies and numerical theories.

**Figure 1:**
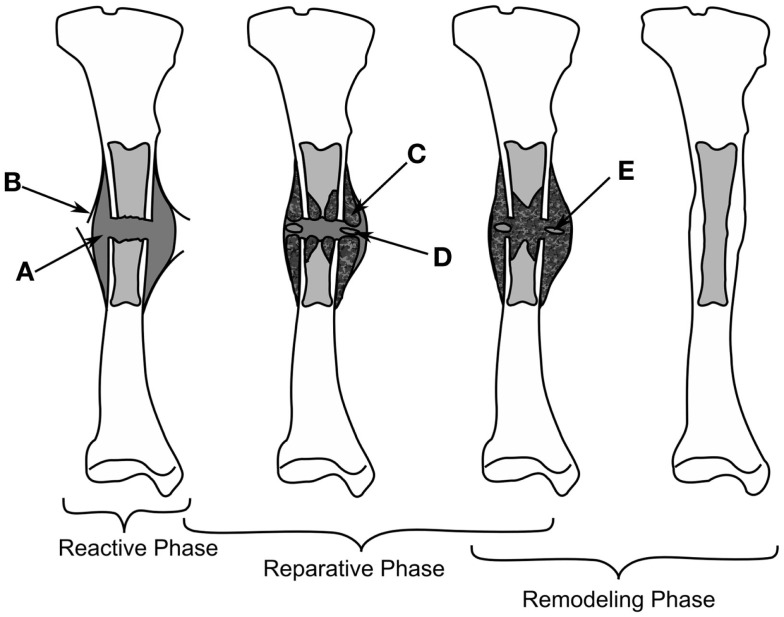
**The healing of a tibial fracture**. A hematoma (A) forms during the reactive phase, beneath the injured periosteum (B). During the reparative phase woven bone (C) forms through intramembranous ossification along with cartilage (C), which is eventually ossified (E), bony bridging occurs and finally the callus is remodeled into cortical bone.

Depending on the stability of the bone fragments the healing can progress down two paths, rigidly fixed fragments with only a small fracture gap can heal through primary bone healing, where the bone remodeling units, responsible for the adaption of the cortical bone, bridge the gap; secondary fracture healing occurs when relative motion occurs between the bone fragments, causing a callus to form. Secondary fracture healing can be divided into three overlapping stages as described in Figure 1, the reactive, reparative, and remodeling phases. Immediately post-fracture there is an inflammatory response, termed the reactive phase, in which blood vessels, which have ruptured fill the injured area with blood forming clot called the fracture hematoma. The fracture hematoma is infiltrated by fibroblasts and small blood vessels, becoming granulation tissue. The initial callus is thus a mixture of hematoma, fibrous tissues, and infiltrating blood vessels. The reparative phase begins once bone and cartilage form, the bone is initially formed through intramembranous ossification initiating on the existing cortical bone and progressing with time toward the plane of the fracture, while the cartilage forms in regions of low oxygen tension (4, 5). Once the blood supply is sufficient enough, cartilage is calcified and converted into woven bone through endochondral ossification. The stability of a fracture influences the amount of intramembranous and endochondral ossification, with more cartilage being formed in less stable fractures and thus more endochondral ossification (6). Bony bridging, the union of the hard callus from either side of the fracture, occurs making the structure extremely stable. The remodeling phase begins during the reparative stage, with the bone structure being adapted back to its original load bearing form. The cortical bone is also remodeled with the cortical bone adjacent to the fracture becoming woven bone, likely as the vasculature within this bone is damaged during the fracture, causing hypoxia (7–9). Hypoxia has been shown to up regulate the formation, size, and activity of osteoclasts, the cells responsible for the resorption of bone (10). The remodeling phase concludes with the callus being completely remodeled into the shape of the original bone, recovering the original strength, and functionality. The events are driven through intercellular signaling, levels of oxygen tension and the mechanical environment directing mesenchymal stems cells to differentiate into osteoblasts, chondrocytes, or fibroblasts each of these cells being responsible for the production of particular tissues (7). In this review, we concentrate explicitly on the mechanical factors influencing bone regeneration, what has been experimentally observed as well as theories and models, which have been developed to explain this.

## Experimental Studies

**Figure 2:**
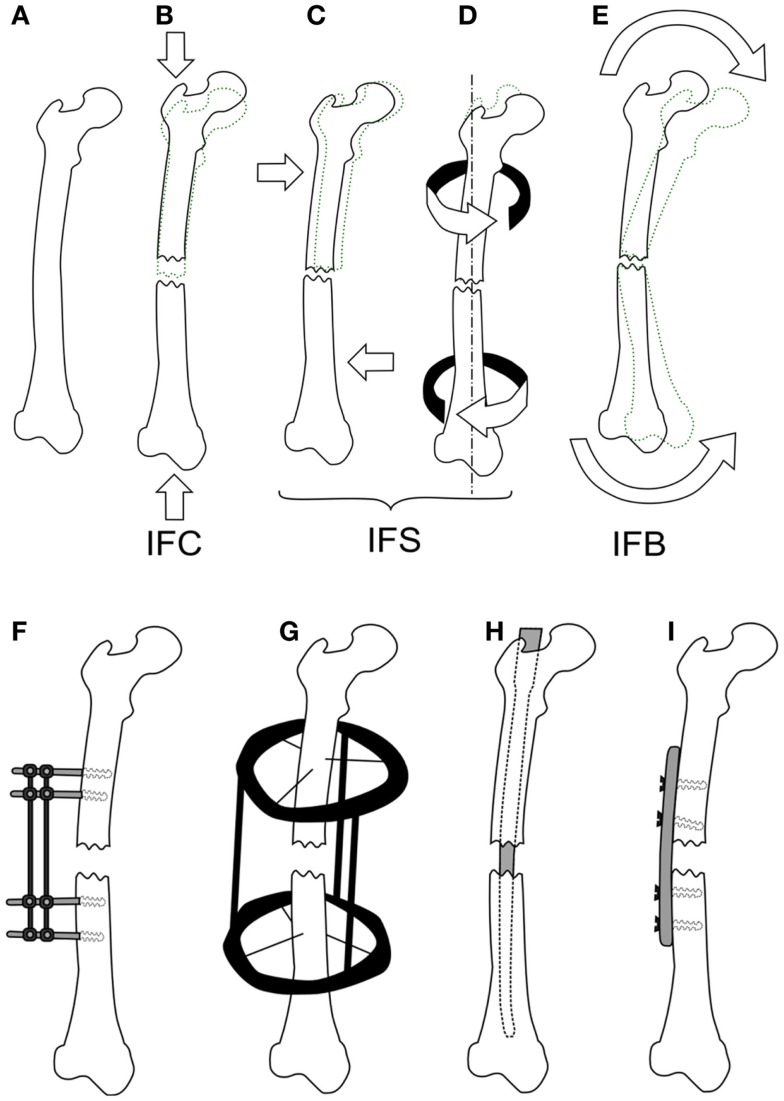
**The different loading modes for a fracture, shown on a femur (A)**. IFC causes in a narrowing of the fracture gap **(B)**, IFS is a shear movement **(C)** across the gap plane, or a relative torsional movement around the axis of the bone **(D)**, and IFB is a bending movement **(E)** centered around the fracture. Fixation methods for fractures are shown in **(F,G)**, external fixator **(F)**, the ring fixator **(G)**, intramedullary nailing **(H)**, and plating **(I)**.

Mechanical loads are applied at the organ level and propagate to a level where cells can sense them, which in turn results in changes at the tissue level. Many studies have investigated applying different forces to fractured bones *in vivo* and quantifying the tissue produced or the mechanical competence of the bone. Loads applied to bone cause non-homogenous strains throughout the healing tissue, resulting in a variety of tissues forming. Experimentally, these strains cannot be quantified *in vivo* preventing the direct development of mechanobiological rules, however, the result of organ level loading is important for validating fracture healing models, as an accurate rule set should predict the outcomes of such studies. Experimental studies investigating the effect of inter-fragmentary movement (IFM) and fixator stability/stiffness are essentially referring to how loading effects fracture healing outcome. IFM is ambiguously used to describe either axial tension/compression of the bone defect, shear movement in the plane of the defect, relative axial rotation of the fragments, or a bending. Here, we will distinguish between these different modes using the following terms: inter-fragmentary compression (IFC), inter-fragmentary tension (IFT), inter-fragmentary bending (IFB), and inter-fragmentary shear (IFS). The different loading modes are illustrated in Figures 2A–E with the exception of IFT, which is just the opposite of IFC. The cases of shear movement and rotation both create a non-uniform shear loading within the tissue, therefore we combine both of these loading states as IFS.

To study the effect of each mode of movement demands the precise control and measurement of bone fragment movement requiring the use of some form of fixation. There are two categories of fixation used in biomechanical research, external and internal fixation, which can be seen in Figures 2F–I. External fixation is commonly used in large animal studies often with modifications to the fixator or to the surgical technique so as to change the stability of the fixator, or be instrumented to measure the fragment displacements (11). In contrast, internal fixators are simpler in design and technique. An intramedullary nail for example is guided by the medullary cavity and can be a single piece, which has made them much more commonly used in small animal studies (12–15). The fixation method has a large effect on the loads, which can be applied, external fixation allows for controlled movements or fixed forces, whereas, internal fixation typically limits the load to an applied force. The fragile nature of the soft tissue means that constant loading throughout a study is not always possible, thus some studies will define a maximum load and displacement. For example, Goodship and Kenwright (16) apply a 33% inter-fragmentary strain or a 360 N load, as initially such a load would induce strains in the hematoma that inhibited healing, whereas, once bony bridging occurs, this level of strain would damage the new bone.

There exists no standard methodology for loading during fracture healing. Studies can either use active loading like Goodship and Kenwright (16) passively allow a limited amount of movement as done by Claes et al. (11) or use a fixator structure or orientation with a different stiffness as done by Klein et al. (17) and Schell et al. (18). While it is often possible to compare initial loading of the callus, the loading is altered as the tissue distribution changes. This can lead to diverging results making comparisons between how bone heals in relation to loading difficult between studies. We attempt to provide a summary of how different organ level loadings affect the healing outcome and were possible highlight variations in fixation and loading between studies.

### Inter-fragmentary compression and inter-fragmentary tension

There are a substantial number of studies, which have investigated the effects of IFC on bone healing, several are summarized within Table 1. It is widely accepted that a certain amount of IFC has a positive effect on the healing process, which was first shown by Goodship and Kenwright (16). The timing of the load is also critical with Gardner et al. (12) showing that immediate application of loading post-surgery resulted in reduced healing potential compared to those applied 4 days after. They also demonstrated that too high an IFC force can be detrimental to healing. However, as they used an intramedullary nail and applied a force rather than a displacement, it is difficult to compare the results. Rate dependence with respect to the application of IFC has been identified by Goodship et al. (19) showing that for the same number of cycles a strain rate of 40 mm/s showed superior healing compared to 2 and 400 mm/s. High frequency low amplitude IFC was investigated by Goodship et al. (20) demonstrating an increase in the callus stiffness.

**Table 1:** **Experimental studies considering the effects of inter-fragmentary compression on fracture healing**.

Author	Study (*n*)	Method	Outcome
Goodship and Kenwright (16)	Sheep (12)	A osteotomy gap of 1 mm in the tibia, fixed with frame fixator, loaded through 33% IFC or 360 N force applied at a frequency of 0.5 Hz	Stimulated callus was significantly stiffer 12 weeks post-surgery compared to rigidly fixed
Claes et al. (11)	Sheep (42)	Six groups with osteotomy gaps of 1.0, 2.0, or 6.0 mm of the tibia and a maximum IFC of 7 or 31%. Fractures fixed with instrumented ring fixator, which measured IFC throughout the experiment	Increased osteotomy gap delayed healing, for 1 mm gap early bony bridging occurred. For larger gaps increased IFC did not enhance healing
Claes and Heigele (4)	Sheep (7)	Osteotomy gap of 3 mm with max allowable IFC of 1.0 mm, fracture fixed with instrumented ring fixator, which monitored IFC over the course of healing. Calcein green injected at 4 weeks and reverin at 8 weeks	IFC reduced over the course of healing. Histological sections appeared to show bone advanced along a path from the cortical surface
Kenwright et al. (21)	Human (85)	Frame fixator applied to tibial fractures, IFC of 0.5–2.0 mm applied at 0.5 Hz for 30 min a day	Group with micro-movements showed a significantly reduced healing time (17.9 vs. 23.2 weeks, *p* = 0.0027)
Kenwright et al. (22)	Human (80)	Frame fixator applied to tibial fractures, IFC of 1.0 mm applied at 0.5 Hz for 30 min a day. Initial loading limited to 12 kg	Healing time to unsupported weight bearing was significantly reduced (23 vs. 29 weeks, *p* < 0.01). Additionally, higher callus stiffness was observed
Gardner et al. (12)	Mice (80)	Tibial osteotomy fixed with an intramedullary nail, loaded with compressive vibrations with a maximum load of 1, 2, and 4 N and amplitudes 0.5, 1, and 2 N where applied. Immediate onset of loading regime was compared to a delayed onset of 4 days	The lowest load case with delayed onset for loading resulted in a significantly higher callus strength. Immediate loading resulted in significantly reduced strength in all cases, and higher loads either in comparable or lower strength
Claes et al. (23)	Sheep (10)	Osteotomy of 2.0 mm mid tibia, two groups 10 and 50% maximum IFC. Fractures fixed with instrumented ring fixator, which measured IFC throughout the experiment. Sacrificed at week 9	Higher IFM resulted in greater fibrocartilage formation, and less bone. No significance in the distribution of blood vessels
Claes et al. (24)	Sheep (10)	Tibial osteotomy of 2.1 or 5.7 mm, both groups had same IFC strain of 30%. Fixation through ring fixator	Larger gap led to fewer blood vessels, less bone formation, and more fibrocartilage
Goodship et al. (19)	Sheep (24)	Mid-diaphyseal tibial osteotomy gap of 3.0 mm, stabilized with a frame fixator. An IFC of 33% or force of 200 N was applied cyclically at 0.5 Hz at strain rates of 2, 40, and 400 mm/s commencing 1 week post-operatively. A secondary study considered the application of the 400 mm/s strain rate 6 weeks post-operatively	The strain rate of 40 mm/s applied 1 week post-operatively showed more mature, stiffer, and stronger callus with a higher BMD when compared to the other groups. There was no significance between 400 and 2 mm/s
Goodship et al. (20)	Sheep (8)	Mid-diaphyseal tibial osteotomy gap of 3.0 mm, stabilized with a frame fixator. IFC was applied at 30 Hz	High frequency loading led to a 3.6-fold stiffer, 2.5-fold stronger, and 29% lager callus compared to controls
Cheal et al. (25)	Sheep (11)	Mid-diaphyseal tibial osteotomy gap of 1.0 mm, stabilized with a flexible pate. A transducer was attached opposite the plate producing a tensile strain gradient from 10 to 100% across the gap	Areas with higher strain led to cortical resorption, while areas with lower strain showed callus development
Mark et al. (26)	Rats (84)	Mid-diaphyseal femoral osteotomy was performed and the gap adjusted from 0–2.0 mm. Axial stiffness was measured at 265 ± 34 N/mm for the 0 mm gap and 30.38 ± 2.07 mm for the 2.0 mm gap	The group with larger gap and less stiffness resulted in a late onset for bone formation and greater endochondral bone formation. Full ossification of the callus was delayed, however, early in the healing stage no difference was found between the two groups histologically
Klein et al. (17)	Sheep (12)	Mid-diaphyseal tibial osteotomy was performed and fixed with a gap of 3.0 mm. The fixation plane varied between the two groups mounted either in the medial plane or anteromedial plane. This lead to differential stiffness between the groups with anteromedial fixation leading to significantly higher IFS and IFC	The group with larger IFM resulted in a stiffer, smaller callus when compared to rigid fixation. The larger IFM group also presented signs of significant remodeling of the callus indicating a more advanced stage of healing

Inter-fragmentary tension is movement applied in the opposite direction to compression, causing an increase in gap size. Cheal et al. (25) showed that high tensile loads lead to reduced healing with even cortical resorption occurring, while lower tensile loads lead to callus formation.

### Inter-fragmentary shear

How IFS affects the bone regeneration process remains controversial, with studies showing that it can inhibit healing and others showing that it can have a positive effect. Several studies in this area are summarized in Table 2, with Bishop et al. (27) and Park et al. (28) showing neutral or positive effects and the others showing negative outcomes. These differences are likely due to the loading conditions. Park et al. (28) studied rabbits in which the femur was fractured instead of the more common method of cutting a discrete unit of bone out with a saw, i.e., osteotomy. They compared compression and shear loading showing an increase in periosteal cartilage formation and a significantly stiffer callus after 4 weeks. It is possible that traumatic injury will solicit a different biological response, which osteotomies do not cause. In addition, fracture planes will not have been as uniform and perhaps will have influenced the tissue loading.

**Table 2:** **Experimental studies considering the effects of inter-fragmentary shear on fracture healing**.

Author	Subjects (*n*)	Method	Outcome
Schell et al. (29)	Sheep (40)	Mid-diaphyseal tibial osteotomy was performed and fixed with a gap of 3.0 mm. Two fixators were used, a rigid fixator and a fixator with high axial rigidity and no resistance to shear motion	The group with free shear movement had significantly reduced torsional strength and stiffness at every time point. Three animals in this group presented hypertrophic non-unions after 6 months
Vetter et al. (9)	Sheep (64)	Mid-diaphyseal tibial osteotomy was performed and fixed with a gap of 3.0 mm. The animals were divided into two groups, one with rigid fixation, and the other with a fixator, which allowed greater shear movement	Histological slices where categorized as belonging to one of six different healing stages based on topological features present. Rigid fixation resulted in a faster progression in healing, this could also be seen in the ratio of bone area to total are which was higher for rigid fixation
Bishop et al. (27)	Sheep (18)	Mid-diaphyseal tibial osteotomy was performed and fixed with a gap of 2.4 mm. Three groups one with rigid fixation, one with torsional shear, and one with IFC. Movement was stimulated to cause 25% principal strain	The group with torsional shear motion had a greater callus area and similar stiffness when compared to the group with no motion, while IFC produced small callus, less advanced with little bridging
Schell et al. (18)	Sheep (64)	Mid-diaphyseal femoral osteotomy was performed and fixed with a gap of 3.0 mm. Two different fixators were used of different stiffness. This resulted in greater IFS within the less stable group	Throughout the healing significantly more cartilage formed with the less rigid fixation group. The rigid group had a larger callus formation. At 9 weeks, there was no significant difference between the two groups
Park et al. (28)	Rabbit (56)	Two cohorts with oblique and transverse tibial fractures each consisting of a rigid fixation and a sliding fixation group. The sliding fixator allowed IFC while the transverse group and IFS in the oblique group	The oblique IFS group showed accelerated healing compared to the other three groups, the torsional strength by 4 weeks exceeded that of intact bone
Klein et al. (30)	Sheep (12)	Mid-diaphyseal femoral osteotomy was performed and fixed with a gap of 3.0 mm. One group of animals was fixed through un-reamed medullary nailing allowing torsional rotation of 10°, the other with a rigid frame fixator. The IFMs were measured throughout	The nailed group showed significantly inferior healing compared to the rigidly fixed group, when comparing mechanical properties and histological sections of the callus after 9 weeks
Lienau et al. (31)	Sheep (64)	Mid-diaphyseal tibial osteotomy gap of 3.0 mm stabilized with a frame fixator. Test group received a fixator, which allowed increased IFS compared to control	Group with higher IFS initially showed a lower blood supply, the healing stage for this group lagged behind, presenting lower stiffness at 6 weeks, this was compensated after 9 weeks. However, the rigid group appeared to have entered the remodeling phase, whereas, the IFS group had not
Epari et al. (32)	Sheep (64)	Mid-diaphyseal tibial osteotomy gap of 3.0 mm, stabilized with a frame fixator. Test group a fixator, which allowed increased IFS compared to control	IFS induced a larger amount of cartilage formation compared control, while also have a more compliant callus. The remodeling process was initiated earlier for rigidly fixed fractures

Bishop et al. (27), for example, applied torsion using a custom designed fixator aiming to produce a principal strain of 25% between the fragments and compared it to equivalent principal strain produced though IFC, he reports torsion having stimulated intercortical mineralization. The complex loading condition presented through shear loading confounds such a comparison, as the pure shear loading produced an equal maximum and minimum principal strains of ±25%, whereas as compression produces a single negative principle strain of −25%. Thus the gap tissue stored twice the amount of elastic energy in the case of IFS as IFC. Additionally, the rotation of 7.2° with a cortical radius of 10 mm and a gap of 2.4 mm would have induced principal strains of 52%, not the 25% stated within the paper. Bishop et al. (27) assumed their torsional fixator to be completely rigid axially with no IFC, in comparison Schell et al. (18) measure both the IFS and IFC for their flexible fixator, when considering just the IFS they calculated a principal strain of ±26%, while including it they received +18.5% and −33%, respectively. It is possible that compliance of the fixator and physiological loading created a beneficial amount of IFC, which was not considered by Bishop et al. (27) in their study. The conclusion which can be drawn is that pure shear motion applied to the whole organ is not pure shear within the healing tissue. It seems inappropriate to compare tissue strain between loading cases using a single value from the strain tensor, a solution would be through using a scalar valued function such as strain energy density (SED), or a combination of deviatoric and volumetric strain.

### Inter-fragmentary bending

The case of IFB has not be sufficiently investigated to form a conclusion, the two studies, which consider this are summarized in Table 3. What has been shown is that asymmetric bending, results in asymmetric callus formation. Healing appears to be inhibited on the tensile side of the callus and promoted on the compressive side (33). This is in agreement with studies looking at axial compression and tension individually as shown before. Cyclic bending appears to cause bone healing to take a different pathway. It has been shown that cyclic bending induces changes in gene expression, where genes responsible for bone morphogenetic proteins are down regulated while genes responsible for cartilage production are up regulated. More cartilage within the callus was observed compared to the unloaded case indicating that the balance of tissue production during the reparative phase was altered (3). The increased level of cartilage indicates that the stimulated callus is not as vascularized as the fixed callus, and the healing will progress along the endochondral ossification pathway rather than through endochondral ossification.

**Table 3:** **Experimental studies considering the effects of inter-fragmentary bending on fracture healing**.

Author	Subjects (*n*)	Method	Outcome
Hente et al. (33)	Sheep (18)	Mid-diaphyseal femoral osteotomy was performed and fixed with a gap of 2.0 mm. Using a custom fixator bending cycles lasting 0.8 s creating a 50% inter-fragmentary strain at the endosteum was applied. The number of loading cycles was varied, the control received no loading, while the first group received 10 bending cycles per day and a second group received 1000 cycles per day	The compressive side of the osteotomy gap resulted in 25-fold greater periosteal callus formation. Greater cycle number showed again a 10-fold difference to the lower cycle number. Bridging occurred exclusively at the compressed side.
Palomares et al. (3)	Rats (85)	Mid-diaphyseal femoral osteotomy of 1.5 mm, the animal were fixed with an external frame, which allowed bending, approximately centered on the gap, the experimental group had stimulated −25/+35° bending applied at 1 Hz for 15 min per day starting 10 days post-surgery	Stimulation up regulated cartilage related genes, and down regulated several genes responsible for bone morphogenetic proteins (BMPs). Serial sectioning showed a much more prolific presence of cartilage and less mineralized callus compared to control.

## Theories and Models

**Figure 3:**
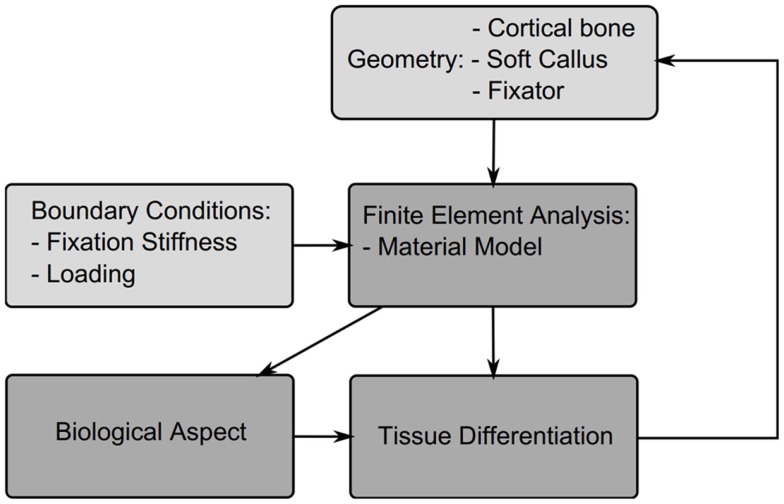
**Structure of a typical fracture healing simulation**. Initially a model is created consisting of the cortical bone fragments, soft callus, and the fixator. Material properties and boundary conditions are then applied to the model based on the tissue distribution and fixator properties, a finite element analysis is performed to determine the mechanicals stimuli, this then is used to drive cell proliferation and tissue differentiation, which updates the tissue distribution and thus new mechanical properties for the next iteration.

In this section, different theories for tissue differentiation are described followed by an overview of the simulations, which have been performed using them (summary of these data can be found in Table 4). Experimental evidence demonstrates that mechanical forces can direct the healing process, i.e., tissue differentiation is mechanobiologically regulated. There are several theories as to which mechanical quantities are the stimuli for differentiation such as SED, deviatoric and volumetric strain, or relative fluid flow between cells and the matrix. Due to the complicated geometries, which occur in fracture healing it is not possible to analytically apply these theories. Instead they are applied as components of simulations, we concentrate here on mechanically driven simulations, which typically consist of five parts summarized in Figure 3; the geometries of bone, defect and callus; the boundary conditions; finite element analysis used to determine the mechanical signal in the callus; the tissue differentiation rules, through which a new callus geometry is created; and finally most simulations consider an additional “biological aspect,” which adds a temporal scale and directs spatially where the ossification occurs. A simulation will go through several iterations changing the tissues composing the callus until a state of equilibrium is reached.

**Table 4:** **Numerical studies**.

Author	Application	Stimuli	Validation/comparison
Huiskes et al. (34)	Bone chamber	Fluid/solid velocity	Søballe et al. (35)
		Shear strain	
Ament and Hofer (36), Palomares et al. (3)	Mid-diaphyseal fracture	Strain energy density	Claes et al. (11)
Lacroix and Prendergast (37)	Mid-diaphyseal fracture	Fluid/solid velocity	Claes et al. (38)
		Shear strain	
Lacroix et al. (39)	Mid-diaphyseal fracture	Fluid/solid velocity	None
		Shear strain	
Bailón-Plaza and van der Meulen (40)	Mid-diaphyseal fracture	Dilatational strains	Goodship and Kenwright (16)
		Deviatoric strains	
Geris et al. (41)	Bone chamber	Fluid/solid velocity	Unpublished pilot study and Geris et al. (41)
		Shear strain	
Shefelbine et al. (42)	Trabecular bone	Dilatational strains	None
		Deviatoric strains	
Kelly and Prendergast (43)	Osteochondral defect	Fluid/solid velocity	None
		Shear strain	
Gomez-Benito et al. (44)	Mid-diaphyseal fracture	Second invariant of deviatoric strain tensor	Claes et al. (38)
Pérez and Prendergast (45)	Bone-implant interface	Fluid/solid velocity	None
		Shear strain	
Isaksson et al. (46, 47)	Mid-diaphyseal fracture	Fluid/solid velocity	None
		Shear strain	
Geris et al. (48)	Bone chamber	Fluid/solid velocity	Geris et al. (48)
		Shear strain	
Chen et al. (49)	Mid-diaphyseal fracture	Dilatational strains	Claes et al. (11)
		Deviatoric strains	
Hayward and Morgan (50)	Mid-diaphyseal fracture, mouse	Fluid/solid velocity	Cullinane et al. (51)
		Shear strain	
Khayyeri et al. (52)	Bone chamber	Fluid/solid velocity	Tägil and Aspenberg (53)
		Shear strain	
Checa and Prendergast (54)	Total hip replacement, stem–bone integration	Fluid/solid velocity	None
		Shear strain	
Isaksson et al. (55)	Mid-diaphyseal fracture	Fluid/solid velocity	None
		Shear strain	
Geris et al. (56)	Mid-diaphyseal fracture	Fluid/solid velocity	None
		Hydrostatic pressure	
Wehner et al. (57)	Tibial fracture	Dilatational strains	Wehner et al. (57)
		Deviatoric strains	
Simon et al. (58)	Mid-diaphyseal fracture	Dilatational strains	Claes et al. (11)
		Deviatoric strains	
Byrne et al. (59)	Tibial fracture	Fluid/solid velocity	Richardson et al. (60)
		Shear strain	
Witt et al. (61)	Tibial fracture	Principal strain with largest absolute value	Witt et al. (61)
Burke and Kelly (62)	Mid-diaphyseal fracture	Substrate stiffness	Vetter et al. (9)
Vetter et al. (63)	Mid-diaphyseal fracture	Various	Vetter et al. (9)
Steiner et al. (64)	Mid-diaphyseal fracture	Dilatational strains	Vetter et al. (9)
		Deviatoric strains	
Steiner et al. (65)	Mid-diaphyseal fracture	Dilatational strains	Epari et al. (66), Bottlang et al. (67), Schell et al. (29), Hente et al. (33), Bishop et al. (27)
		Deviatoric strains	

The callus geometries are typically assumed to be constant and ellipsoidal, so simulations of fracture healing start at the reparative phase once the soft callus has formed. The only exception to this is the model of Gomez-Benito et al. (44) who presented a model, which allows the callus boundaries to evolve over time. Boundary conditions are based upon the loading described by the experimental study the authors aim to replicate.

### Tissue differentiation theories

In 1960, Pauwels first proposed that tissue differentiation within a fracture callus was governed by mechanical stimuli. He theorized that cartilage formed as a result of local hydrostatic pressure causing mesenchymal stem cells to become chondroblasts, whereas, bone and fibrous tissues resulted from shear strains causing mesenchymal stem cells to differentiate into osteoblasts and fibroblasts, respectively (68). Perren and Cordey (69) defined the upper limits of mechanical stimulation of fracture healing. Their inter-fragmentary strain theory states that the tissue within the fracture gap must be capable of withstanding the strain produced by the IFM. They then suggested that rigid fixation of fractures should result in the healing process commencing at a later stage. This was later contradicted by the study of Goodship and Kenwright (16) that showed that a certain level of micro-movement accelerated aspects of the healing process. However, their inter-fragmentary strain theory certainly governs what tissues can exist and is particularly important in cases where tension is dominant.

The theory of Pauwels (68) was numerically investigated by Carter et al. (70). As a fracture callus is an internal three dimensional structure, it was not possible to study the strain *in vivo*. Using a finite element model of an idealized fracture geometry with soft callus they investigated what they called the osteogenic index, a relationship between hydrostatic pressure and octahedral shear stress.

$$I=\sum_{i=1}^{c}n_{i}\left(S_{i}+kD_{i}\right)$$

Where *I* is the osteogenic index, *c* is the number of load cases, *n* is the number of loading cycles, *S_i_* is the cyclic octahedral shear stress, *D_i_* is the cyclic hydrostatic pressure, and *k* is a scaling factor relating the two. They also recognized that the load applied to a fracture would not be constant, but vary between different load cases. The osteogenic index was therefore a summation of the mechanical signals at these different load cases. The proposed theory also considered a distinction between tissues with poor and good bloody supply, with good blood supply being capable of forming all tissue types, but requiring significant hydrostatic pressure to form cartilage, whereas, tissue with poor blood supply formed either connective tissue of cartilage (70).

**Figure 4:**
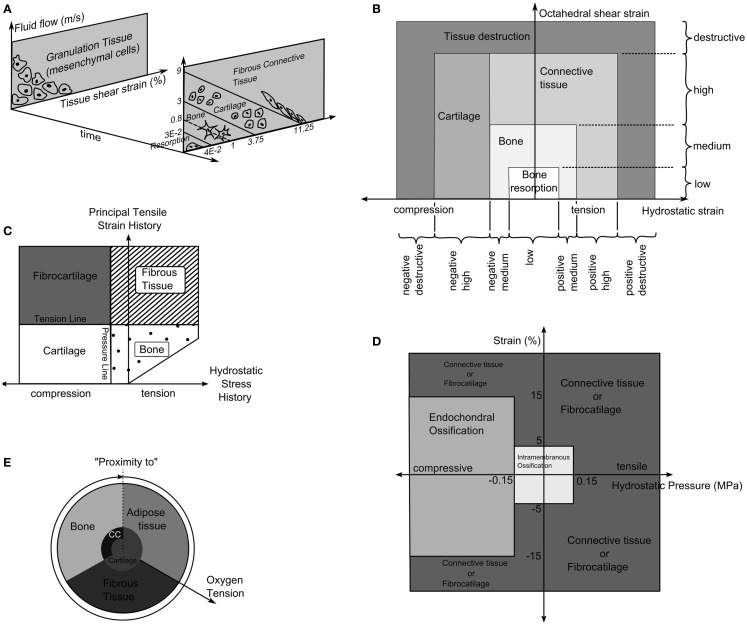
**(A)** The tissue differentiation rules based on fluid flow relative to solid phase and shear strain. Reprinted from Lacroix and Prendergast (37) with permission from Elsevier. **(B)** Tissue differentiation based on hydrostatic and octahedral shear strain. Reprinted from Shefelbine et al. (42) with permission from Elsevier. **(C)** The tissue differentiation rules with pressure line and tension line. Reprinted from Carter et al. (73) with permission from Lippincott Williams and Wilkins. **(D)** The tissue differentiation rules using hydrostatic pressure and strain. Reprinted from L. Claes and Heigele (4) with permission from Elsevier. **(E)** The tissue differentiation rule based on substrate stiffness and oxygen tension. Reprinted from Burke and Kelly (62) with permission from PLoS ONE.

Prendergast and Huiskes (71) studied how the osteogenic index differed between the use of linear-elastic or poro-elastic material properties for the healing tissues. The aqueous nature of biological tissues, particularly soft tissues, means that representing them as a mixture of fluid and solid phases describes the tissue behavior more accurately than linear-elastic models. Using the experiments of Søballe et al. (35), where a loadable bone chamber was implanted in the femoral condyle of canines and the tissues, which formed under different loadings quantified cross-sectionally over a number of weeks, Prendergast and Huiskes (71) were able to determine that the poro-elastic model predicted the osteogenic index more appropriately when compared to tissue distributions in the experiment. They expanded on this work by developing a new theory, that the relative velocity between fluid, solid, and shear strain, rather than hydrostatic pressure and shear strain where the stimuli for tissue differentiation as described by Figure 4A (72).

$$\begin{array}{c} S=\gamma ∕a+\nu ∕b\\ \begin{array}{cc} \mathrm{Condition}\;\mathrm{for}\;\mathrm{bone}: & S<S_{\mathrm{bone}}\\ \mathrm{Condition}\;\mathrm{for}\;\mathrm{cartilage :} & S_{\mathrm{bone}}<S<S_{\mathrm{cartilage}}\\ \mathrm{Condition}\;\mathrm{for}\;\mathrm{fibrous}\;\mathrm{connective}\;\mathrm{tissue}: & S>S_{\mathrm{cartilage}} \end{array} \end{array}$$

where γ is the deviatoric shear strain, ν is the solid/fluid velocity, and *a* and *b* are empirically derived constants varying for each tissue type.

The relationship was defined as a summation of maximal distortional strain and relative velocity between fluid and solid (34). It is important to reiterate that this theory was developed from experiments using a bone chamber, which has a simple geometry known *a priori* that can be easily represented in finite element simulations with the applied loads being known. In contrast, the boundaries of the callus are not known exactly and the loading is an approximation of the physiological condition.

Carter et al. (73) proposed that tissue differentiation was determined through tensile principal strain and hydrostatic pressure as shown in Figure 4C. High principal tensile strains result in fibrous tissue when pressure is low and fibrocartilage when pressure is high, whereas, low principal tensile strains results in bone and cartilage when pressure is low or high, respectively.

The theory of Pauwels (68) was revisited by Claes et al. (38) who in a large interdisciplinary study compared the tissue distribution of healing tibial fractures in an animal study and equivalent finite element study. They attempted to determine the values of hydrostatic pressure and axial strains, which caused differing tissue differentiation. They determined that bellow a hydrostatic pressure of 0.15 MPa and strain <5% stimulate intramembranous ossification, compressive hydrostatic pressure >0.15 MPa and strain <15% stimulated endochondral ossification and that pressure and strain outside of these regions resulted in fibrous tissue or cartilage, as shown in Figure 4D. Claes and Heigele (4) then proposed ossification only occurs on an existing bony surface. While they did not develop a simulation, they created FE models, which represented the healing callus at different stages and correlated the stress and strain from these models with histological sections from an animal study, which was performed in parallel.

Ament and Hofer (36) presented a simulation using a fuzzy logic controller with nine linguistic rules, which defined how levels of SED and the concentration of bone in neighboring elements to control the differentiation of elements into three tissue types, cartilage, bone, and fibrous connective tissue. The SED could be within four different levels; low, physiological, increased, and pathological. These were independent of the tissue type, as SED is relatively invariant to tissue type. Results were compared with the experiments of Claes et al. (11) and showed strong similarities in the reduction of IFM over time.

Fuzzy logic was again used by Shefelbine et al. (42), modeling trabecular regeneration. Their model was based on the proposed relationship of Claes and Heigele (4). However, they modified the tissue differentiation theory replacing the hydrostatic pressure criteria with an equivalent volumetric strain. Thus, the mechanical stimuli became volumetric strain and octahedral shear strain, described in Figure 4B. This model had 21 linguistic rules to describe how tissue differentiated; it considered three tissue types, bone, cartilage, and fibrous tissue. In addition, vascularization was also modeled using fuzzy rules. The simulation required the strains in a particular element to reach a certain range before tissue within the element began differentiating, for bone to form the elements must also have sufficient vascularity while cartilage formed independently of vascularity. The vasculature was also driven mechanically only advancing to elements within an acceptable strain level. The model was implemented as a three dimensional linear-elastic simulation. While this model can capture the events of bone regeneration, Steiner et al. (65) conducted a parameter study of fixator stiffness, which encompassed values used several *in vivo* studies and achieved comparable outcomes, it is a linguistic representation of observed phenomenon. Thus it is entirely phenomenological and does not encompass any underlying mechanism in physical or chemical terms. This raises issues with regards scaling, should the resolution of the model or length of the iteration change, as there is no governing equation.

## Biological Aspects

The majority of simulations included an additional level often described as the biological aspect. As Claes and Heigele (4) observed the front of healing bone follows a path, starting at the original cortical faces and advancing toward the fracture gap. The inclusion of biological aspects in the form of cell, revascularization, nutrient supply, or oxygen tension, allows ossification to follow such a path. These biological elements are typically modeled as diffusive processes (39), random walks (45, 74), or through logical association of neighboring elements (36, 42). While cell proliferation and revascularization are vital aspects of fracture healing, the interplay between mechanical forces and these processes are not fully understood, in addition there is limited experimental data available so the validation of such model becomes much more difficult.

How the biologic aspect influences the course of fracture healing can vary, Lacroix et al. (39) considered cells diffusing within the callus and scaled the tissue stiffness according to cell destiny within an element. An issue exists with this approach, as the maturity of the tissue is determined purely by the cell number within and the tissue phenotype by the mechanical stimuli this allows mature cartilage to switch directly to mature bone. Kelly and Prendergast (43) similarly applied a scaling, but considered multiple cell phenotypes and so multiple tissues within a single element removing this issue. Shefelbine et al. (42) considered nutrient supply to be the critical biological factor in bone development, and so bone could only form in areas with good vascularization. This was again used by Wehner et al. (57) and Simon et al. (58). Chen et al. (49) added an additional level to this considering nutrient diffusion from the developing vasculature.

Burke and Kelly (62) proposed a theory in which tissue differentiation is indirectly driven by mechanical forces. Revascularization is allowed on in elements where the deviatoric strain is below a level of 6%. The blood vessels were assumed to diffuse into the tissue.

$$\underset{\Omega_{\gamma <0.06}}{\int }\frac{dV}{\mathrm{dt}}\mathrm{dx}=\underset{\Omega_{\gamma <0.06}}{\int }0.5\times \Delta V\mathrm{dx}$$

Where *V* is the vascularity, γ is the deviatoric strain, and Ω is the computational domain with all elements where this strain was <6%. The oxygen is then assumed to diffuse from the vasculature without any dependence on the local mechanical environment.

$$\underset{\Omega }{\int }\frac{dO_{2}}{\mathrm{dt}}\mathrm{dx}=\underset{\Omega }{\int }D\Delta O_{2}-Q\cdot n^{\max}n\;\mathrm{dx}$$

Here *O*_2_ is the oxygen concentration, *D* is the diffusion coefficient of oxygen in the tissue, *Q* is the oxygen consumption rate of cells in the tissue, and *n* represents the number of cells in the element and *n*^max^ the maximum cell density. The tissues then differentiated based on the oxygen tension and the stiffness of neighboring elements as described in Figure 4E.

Several models exist which consider purely biological factors in fracture healing, Bailon-Plaza and Van Der Meulen (75) first proposed a model for bone regeneration, which included diffusion of stem cells, and various growth factors, but no mechanical feedback. Geris et al. (76) applied this model to simulate the healing of tibial fractures in mice, finding that the model could predict the course of healing, but was sensitive to initial levels of growth factor production. Geris et al. (56) investigated if mechanical regulation of angiogenesis and growth factor production could improve this model and account for load induced non-unions, and concluded that mechanical feedback for both angiogenesis and osteogenesis was required to correctly predict unions and non-unions. Later changes to this model have excluded mechanics and focused on more detailed representations of angiogenesis (77, 78).

These theories consider cell density in homogenous tissue elements and apply rules for motion, differentiation, and proliferation to the cell population based upon tissue level stimuli. *In vivo*, the structure of tissues within callus is microscopically heterogeneous, thus mechanical stimuli at the tissue level are not easily translated to the cellular level. While at the tissue level, strains and perfusion of interstitial fluid (ISF) in the callus can be accurately determined, the stimulation they cause at the cellular level will be different for each cell within a tissue element due to the heterogeneity. This has not yet been quantified *in vivo*, however, fluid dynamic studies of perfusion bioreactors can lend insights as to the heterogeneity of mechanical stimuli when fluid is perfused through a structure at physiological rates. Zermatten et al. (79) used high resolution micro computed tomography (micro-CT) images of bone tissue engineering scaffolds. Fluid dynamic simulations of medium perfusion showed the wall shear stress within a scaffold had a wide range of values. It has been known for some time that *in vitro* osteoblastic differentiation and bone formation are augmented with ISF flow (80), however, application of this biological information *in silico* will require more detailed models of the tissue structure.

The features all these biological aspects share are the boundary conditions, considering the periosteum of the cortical fragments and the surrounding tissue at the source of cells, nutrients, or vascular tissue. The propagation of these are mainly all diffusive processes, though for ease of implementation and to reduce computational power required implementations have varied, the random walk used by Pérez and Prendergast (45) and fuzzy logic of Shefelbine et al. (42) allowed diffusive behavior without the computational cost of solving the diffusion equation numerically.

### Comparison of model performance

**Figure 5:**
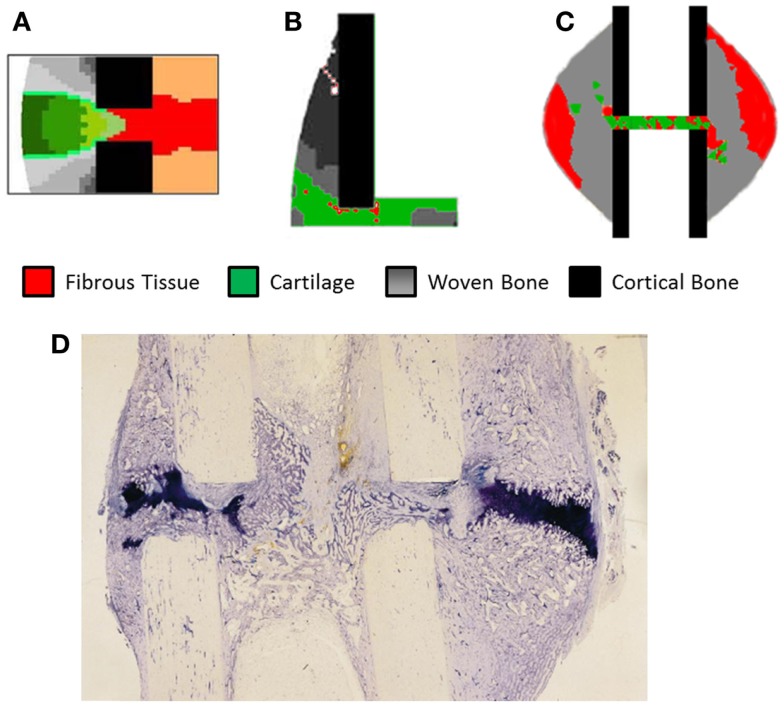
**The results of fracture healing simulations, (A) Burke and Kelly (62) reprinted with permission from PLoS ONE**. **(B)** Lacroix and Prendergast (37) reprinted with permission from Elsevier. **(C)** Steiner et al. (65) reprinted with permission from PLoS ONE. **(D)** Histological section of healing ovine tibia, new woven bone is lightly stained, while cartilage is darkly stained. Reprinted with permission from Elsevier.

Validation of models has remained a significant problem, looking at Table 4 approximately half of the studies have no experimental reference. The study of Claes et al. (11) has frequently been used as a comparison due to clear experimental method and inclusion of IFC values longitudinally as well as histological slices obtained cross-sectionally. However, while the IFC data are available no quantitative comparison has be made between simulations and the results, instead visually comparing general trends and histological slices has predominated. In Figure 5, we see simulation results from (A) Burke and Kelly (62), (B) Lacroix and Prendergast (37), and (C) Steiner et al. (65) these all consist of a uniform cortical bone and callus geometry, which is made up of non-uniform finite elements, in the case of Burke and Kelly (62) and Lacroix and Prendergast (37) they are two dimensional axisymmetric simulations and in Steiner et al. (65) three dimensional. Figure 5D is a histological section from an *in vivo* study by Claes and Heigele (4); we see the callus is non-uniform and asymmetric. This mismatch between the asymmetric callus in the histological images and uniformly simulated calluses complicates direct comparison, additionally axisymmetric boundary conditions implies the simulation should match every quadrant of the histological slice, which is clearly impossible, when using three dimensional models like Steiner et al. (65) one must also find the correct slice of the model to compare to the histology. Vetter et al. (63) compared the use of volumetric strain, deviatoric strain, greatest-shear strain, and principal strain as stimuli for tissue differentiation. Through carrying out a parametric study they found that all of these could accurately predict bone healing within a range of thresholds. They used quantitative metrics to assess the accuracy, comparing averaged histological sections from Vetter et al. (9) with simulated images. This allowed the comparison of volume fraction and number of co-located pixels. However, this work was in two dimensions and thus did not consider IFS or IFB. The data of Vetter et al. (9) were later used a visual comparison by Burke and Kelly (62), who showed their simulations appeared to agree well with the results, however, no quantitative comparison was used.

There are several studies comparing the performance of the different tissue differentiation theories. Isaksson et al. (81) and Epari et al. (82) both compared the models of Prendergast and Huiskes (71) and Claes and Heigele (4). Both used three dimensional models with poro-elastic element properties, however, different results were presented for similar load cases. Isaksson et al. (81) found that under 7.2° of torsion only the model of Prendergast and Huiskes (71) correctly predicted healing. While Epari et al. (82) found that for 10° of torsion both models predicted fracture healing. The differences can possibly be explained through differences in material properties, with Isaksson et al. (81) using an elastic modulus for the granulation tissue an order of magnitude larger than Epari et al. (82). However, the mechanical properties for all of the tissues included by Epari et al. (82) are not listed, making a complete comparison not possible. One further erroneous element is the formation of unconnected bone in both studies when using the Claes and Heigele (4) differentiation theory. This theory explicitly states that ossification occurs in the soft tissue contacting the surface of existing bone, and that the rest of the callus is fibrous tissue unless a pressure of −0.15 MPa is present and then it becomes cartilage. The formation of unconnected bone can only imply that both implementations did not consider this aspect of the differentiation rules, and applied the surface ossification rules throughout the callus. To complicate this further Klein et al. (30) and Bishop et al. (27) found contradictory results for healing under torsional loading. The theory of Shefelbine et al. (42) is essentially an equivalent theory to Claes and Heigele (4) except it uses deviatoric and dilatational stress and strain rather than stress. This model has been used by Steiner et al. (64), Wehner et al. (57), and Simon et al. (58). Steiner et al. (64) demonstrated that with the correct threshold values the model could predict union for the cases of IFC and IRF, but non-union for IFS.

Isaksson et al. (83) again compared the theories of Claes and Heigele (4) and Prendergast and Huiskes (71), as well as Carter et al. (73). They found the theories predicted extremely similar temporal and spatial patterns in healing. In addition, they compared the volumetric components (relative fluid solid velocity and pore pressure) of the theories with the distortional components, finding that deviatoric strain alone could also predict similar tissue formations, whereas, volumetric components such as pore pressure and fluid velocity could not predict healing by themselves. This is however contradicted by Isaksson et al. (81) who found that the tissue formation predicted by deviatoric strain alone did not match *in vivo* data.

Hayward and Morgan (50) implemented a model using Prendergast and Huiskes (71) theory of tissue differentiation. They used three dimensional models based on micro-CT images of a mouse femur. The model correctly predicted a mechanically induced non-union and larger volume of cartilage formed due to the loading, but was not completely accurate in the patterns of tissue formation, predicting an excess of bone formation within the gap. Checa et al. (84) investigated if the Prendergast and Huiskes (71) theory could be directly applied different species, specifically rats and sheep. They reported that differences in healing between the species observed *in vivo* cannot be purely attributed to differences in loading and animal size, but also the thresholds for formation are different between, for sheep a larger mechanical stimuli was capable of forming bone, which in the case of the rat would result in cartilage. Given that Hayward and Morgan (50) did not scale these parameters for mice, this is a limitation of their study, which was not considered.

Another form of validation is possible; instead of using results directly from fracture healing one can compare how algorithms perform when modeling a bone chamber. Geris et al. (48) compared the differentiation rules of Prendergast and Huiskes (71), Carter et al. (73), and Claes and Heigele (4) against tissue formation in a bone chamber. They concluded that the models only partially matched experimental findings, with models predicting cartilage formation, which was not observed experimentally. The implementation of Prendergast and Huiskes (71) algorithm did not consider bone resorption, which is found in the implementation of Lacroix and Prendergast (37). As the other algorithms do not include resorption this modification is understandable to enable comparison between them, however, it would be interesting to see if its inclusion increased or decreased the accuracy.

## Outlook

Mechanical forces and bone regeneration are intrinsically linked. While there has been a vast amount of research on this topic, experimental comparison between studies is extremely difficult. The greatest difficulty comes from non-standard experimental setups, specifically, different defect sizes and use of modified and non-standard fixators. While great efforts have been made to characterize the movements and stiffness of devices, without knowing the true bone geometry and exact tissue composition it is difficult to determine the precise strains tissues are experiencing. These factors play a part in the validation of simulations using this data, as differences in geometry between simulation and experiments should cause different mechanical stimuli.

Validation of models with experimental data is crucial for all simulation work, and is the distinguishing factor between simulation and animation. What is clear is that in the field of bone regeneration, comparing the results of *in silico* studies with *in vivo* studies is not trivial. It is impossible to measure every factor simultaneously, yet models for fracture healing have become increasingly complicated. More factors are considered such as cellular events, revascularization, and even protein secretion. While validation of such models is not impossible it requires large cross-sectional studies, which have yet to be performed. To compound this, current models of bone regeneration all consider tissues as homogenous continuum, while woven bone is a macroscopically porous structure. The theories of Carter et al. (73), Claes and Heigele (4), and Prendergast et al. (72) are based on comparing the results of continuum finite element analyzes with histological data, and correlating the areas where bone has formed with the local mechanical environment. The histological data were the result of cross-sectional studies, so while they could observe the formation of woven bone, they could not see how individual structures evolved. One solution to this problem lies in *in vivo* micro-CT. Micro-CT uses two dimensional X-ray images of a sample taken from multiple directions to reconstruct a three dimensional image of the sample. The application of *in vivo* micro-CT means time-lapsed longitudinal studies can be performed, where the same animals can be imaged at several time points. These images can then be compared, highlighting the changes in the regenerating tissue. When combined with a well-defined loading regime an equivalent finite element analysis can be performed using the micro-CT images as a basis. The results of which can be compared to the changes in tissue observed *in vivo*, allowing the validation or falsification of current theories for tissue differentiation.

The development and maturation of the callus (reactive and reparative phase) is a single aspect of fracture healing, eventually the bone must remodel to its original form, while Lacroix and Prendergast (37) include this, it has never been shown that this is the correct mechanism. Schell et al. (8) have shown that early in the healing process osteoclasts are present, and that cortical bone closest to the defect gets remodeled into woven bone, then back to cortical. With i*n vivo* micro-CT based studies this remodeling can be quantified. With quantitative data remodeling theories can be corroborated. Such a study could provide a window into the relationship between cortical remodeling and trabecular remodeling and what causes the differentiation between the two.

Subject specific simulations are a necessity, allowing direct comparison between simulations and experimental results on a sample by sample basis. Currently, no study has performed an animal specific simulation and determined if the simulation produces results without a significant difference to the *in vivo* results. This will remove the any error associated with averaging experimental results so they can be compared to simulations using idealized geometry. Additionally simulations using realistic geometry and loading conditions will allow the effects of the non-uniform bone geometry and strain distribution within the callus to be quantified. In Figure 5D, we can see clearly how asymmetric the callus is, and that on the left side of the bone cartilage has formed within the osteotomy gap whereas on the right side of the image the cartilage is bridging the larger hard callus. The asymmetry of the bone, callus, and loading cannot be captured in axisymmetric models and only partially using idealized models of the geometry. This geometric information will be the product of studies using *in vivo* micro-CT. What parameters should be compared between such models is an open question, which must be addressed first. Work already presented in the field of bone remodeling and adaptation (85) provides a basis for what can be measured, and models for bone remodeling such as Schulte et al. (86) may also be incorporated in simulations, representing the remodeling phase.

## Conclusion

This review has presented how the mechanical models and experiments of fracture healing have developed since Pauwels (68) first proposed his theory. The differences between the types of data produced by simulations and experimental studies remains an obstacle for advancing the field. Existing rules without exception have all been derived from two dimensional continuum finite element models, though associating the simulated mechanical stimuli to tissue growth seen in histological section from cross-sectional a study. As more detailed, quantitative and longitudinal data are being gathered experimentally these rules must be re-examined, their accuracy assessed using longitudinal time-lapsed data. With simulations there is a need for simulations to move away from simplified representations of the geometry with continuum material properties, toward real bone microstructural geometries measured through micro-CT, so as to allow direct and quantitative comparison of their predicted tissue distribution directly to the results of *in vivo* studies, rather than a visual comparison with single histological slices.

## Conflict of Interest Statement

The authors declare that the research was conducted in the absence of any commercial or financial relationships that could be construed as a potential conflict of interest.
